# Mesenchymal stem cells - the secret agents of cancer immunotherapy: Promises, challenges, and surprising twists

**DOI:** 10.18632/oncotarget.28672

**Published:** 2024-11-22

**Authors:** Theia Minev, Shani Balbuena, Jaya Mini Gill, Francesco M. Marincola, Santosh Kesari, Feng Lin

**Affiliations:** ^1^CureScience Institute, San Diego, CA 92121, USA; ^2^Sonata Therapeutics, Boston, MA 02472, USA; ^3^Department of Translational Neurosciences, Pacific Neuroscience Institute and Providence Saint John’s Health Center, Saint John’s Cancer Institute, Santa Monica, CA 90404, USA

**Keywords:** mesenchymal stem cells, genetic engineering, cancer immunotherapy, mesenchymal stem cell homing, stem cell delivery

## Abstract

Mesenchymal stem cells (MSCs) are recognized for their immunomodulatory capabilities, tumor-homing abilities, and capacity to serve as carriers for therapeutic agents. This review delves into the role of adoptively transferred MSCs in tumor progression, their interactions with the tumor microenvironment, and their use in delivering anti-cancer drugs, oncolytic viruses, and genetic material. It also addresses the challenges and limitations associated with MSC therapy, such as variability in MSC preparations and potential tumorigenic effects emphasizing the need for advanced genetic engineering and personalized approaches to enhance therapeutic efficacy. The review concludes with an optimistic outlook on the future of MSC-based therapies, underscoring their promise to develop effective and personalized cancer treatments.

## INTRODUCTION

Cancer immunotherapy has revolutionized the treatment of various malignancies by harnessing the body’s immune system to fight cancer. Traditional cancer therapies, such as chemotherapy, pathway inhibitors and radiation, directly target tumor cells but often come with significant side effects and resistance issues. Moreover, these therapies are intended to exploit a single or a few anti-cancer mechanisms. Since cancer biology is a rapidly evolving evolutionary process, the limited scope of most therapies allows for the development of immune escape and resistance to therapy. In contrast, immunotherapy aims to enhance or restore the immune system’s ability to recognize and eliminate cancer cells, which in turn when successful results in the activation of multiple anti-cancer mechanisms that reduce the chances of escape [1]. This approach offers a more targeted and potentially less toxic alternative to conventional treatments, leveraging the body’s natural defenses to combat cancer. Immunotherapy approaches include immune checkpoint inhibitors [2], chimeric antigen receptor (CAR)-T cell therapy [3, 4], cancer vaccines [5], and monoclonal antibodies [6]. These immunotherapies have shown remarkable efficacy in hematologic malignancies and in a subset of solid tumors. CAR-T cell therapies and immune checkpoint inhibitors have led to impressive response rates and durable remissions in patients with refractory or relapsed blood cancers. Immune checkpoint inhibitors have shown efficacy in treating melanoma and non-small cell lung cancer (NSCLC), with pembrolizumab becoming a first-line treatment for PD-L1-positive NSCLC. Moreover, research is ongoing to identify suitable targets and develop effective immunotherapies for a broader range of solid tumors, including colorectal, breast, and pancreatic cancers. Combining immunotherapy with other treatment modalities, such as chemotherapy, radiation, and targeted therapies, is being actively explored to enhance treatment outcomes [7].

Mesenchymal stem cells (MSCs) are multipotent stromal cells that can differentiate into various cell types, including osteoblasts, chondrocytes, and adipocytes. They are identified by a specific set of surface markers, most commonly CD73, CD90, and CD105, while lacking the expression of CD45, CD34, CD14 or CD11b, CD79alpha or CD19, and HLA-DR surface molecules [8]. MSCs are primarily isolated from bone marrow, but they can also be derived from other tissues such as adipose tissue, umbilical cord blood, placenta, and dental pulp, offering additional avenues for harvesting these cells with minimal ethical concerns [9]. The appeal of MSCs in therapeutic applications stems from their dual capabilities of self-renewal and differentiation. This means they can not only produce identical daughter cells to maintain the stem cell pool but also generate specialized cells that contribute to tissue repair and regeneration [10]. Furthermore, MSCs possess significant immunomodulatory properties, allowing them to modulate immune responses in a variety of ways. They achieve this through direct cell-to-cell contact and the secretion of a broad range of bioactive molecules, including cytokines, chemokines, and growth factors [11].

This review discusses MSCs’ immunomodulatory properties, their use as carriers for therapeutic agents, and their inherent homing abilities, alongside genetic engineering to enhance their therapeutic efficacy. We also summarize the key preclinical studies and relevant clinical trials, outlining the challenges, limitations, and future directions of this promising cancer treatment approach.

## MSCs AND THEIR ROLE IN MODULATING THE IMMUNE SYSTEM AND TUMOR PROGRESSION

MSCs are being increasingly recognized for their ability to modulate the immune system, which can significantly affect tumor progression. Their immunomodulatory effects are mediated through both direct cell-cell contact and the secretion of various soluble factors. MSCs can create an immunosuppressive microenvironment that may either support tumor growth or inhibit immune responses against the tumor [12].

MSCs interact directly with various immune cells through cell surface molecules and receptors. For instance, MSCs express Programmed Death-Ligand 1 (PD-L1), which can bind to the PD-1 receptor on T cells, leading to T cell inactivation [13]. This interaction is pivotal in reducing the proliferation and cytotoxic activity of T cells, thus contributing to an immunosuppressive environment.

MSCs secrete a diverse array of cytokines, chemokines, and growth factors that modulate immune responses, including (i) Transforming Growth Factor-beta (TGF-β), which suppresses T cell proliferation and promotes the development of regulatory T cells (Tregs), (ii) Interleukin-10 (IL-10), which inhibits the activity of pro-inflammatory cytokines and immune cells, fostering an anti-inflammatory and immunosuppressive milieu, (iii) Indoleamine 2,3-dioxygenase (IDO), which depletes tryptophan in the local microenvironment, leading to T cell anergy and apoptosis, thereby dampening the immune response, (iv) Prostaglandin E2 (PGE2), which modulates the function of dendritic cells, T cells, and NK cells, further contributing to the suppression of immune responses [14, 15].

Therefore, MSCs can create an immunosuppressive microenvironment to inhibit immune responses against the tumor. In the tumor microenvironment, MSCs can promote tumor progression by enhancing the recruitment of regulatory T cells (Tregs) and myeloid-derived suppressor cells (MDSCs), as well as by promoting angiogenesis through the secretion of vascular endothelial growth factor (VEGF) and other pro-angiogenic factors, which support tumor growth and metastasis [16].

**Figure 1:**
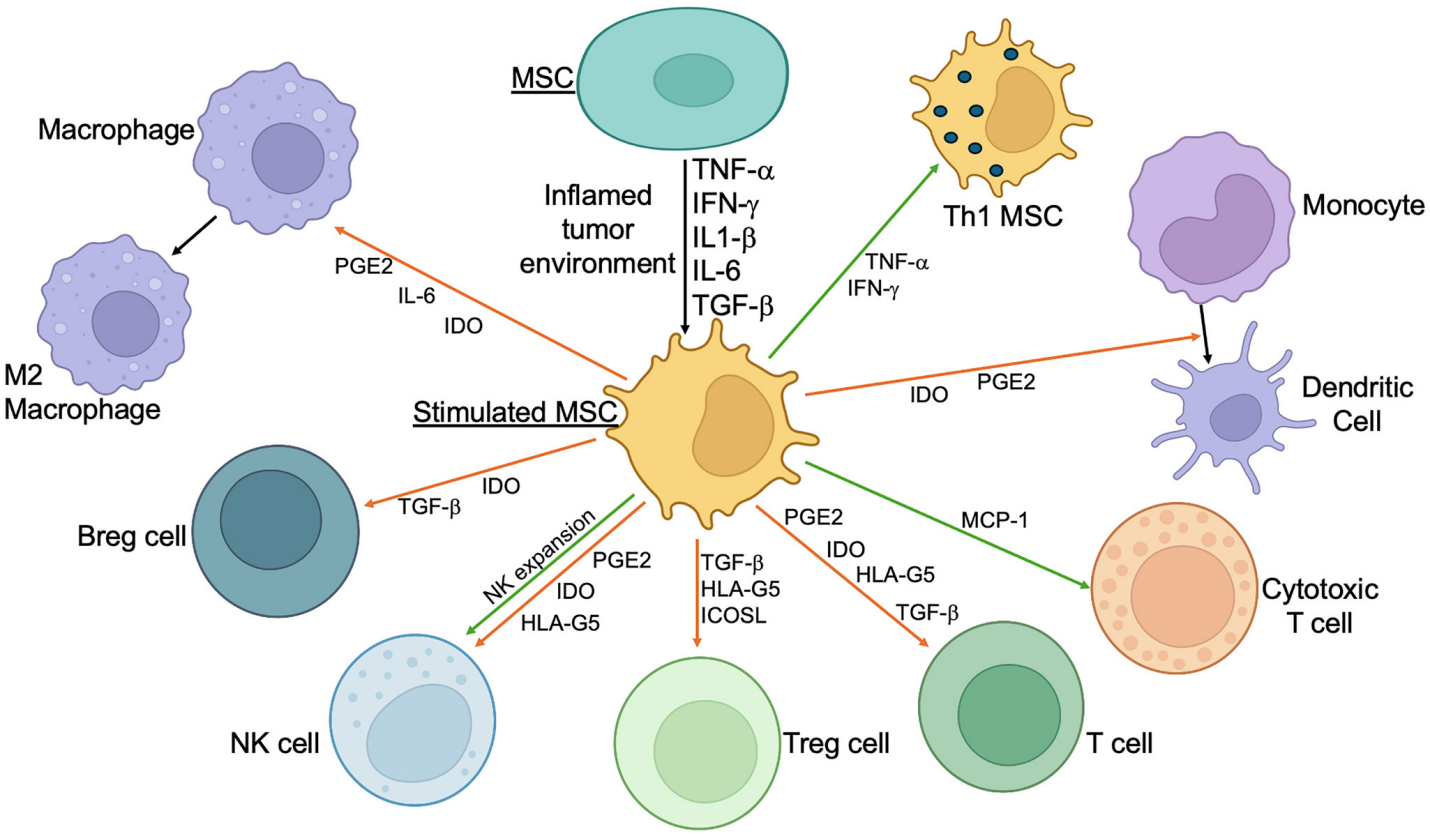
Immunomodulatory effects of simulated MSCs. MSCs utilize different molecular mechanisms to suppress (in most cases) or activate immune cells. Orange arrows: inhibition; Green arrows: stimulation. Abbreviations: INF-γ: interferon gamma; TNF-α: tumor necrosis factor alpha; IL-1β: interleukin-1 beta; PGE-2: prostaglandin E2; IDO: indoleamine 2,3-dioxygenase; TGF-β: transforming growth factor-β; HLA-G5: human leukocyte antigen-G5; Treg: T regulatory lymphocytes; Breg: B regulatory lymphocytes; IL-6: interleukin 6; MCP-1: Monocyte Chemotactic Protein 1; Th1: T helper type 1; ICOSL: Inducible T-cell co-stimulator ligand; NK: Natural killer cell.

However, under certain conditions, MSCs can also enhance anti-tumor immunity by modulating the activity of immune cells. For example, MSCs can support the expansion of large numbers of NK cells with an elevated cytotoxicity profile [17]. In some contexts, MSCs can promote the activation and proliferation of effector T cells, thereby enhancing the immune response against tumors [18]. Moreover, the presence of IFN-g and TNF-a can polarize immune suppressive MSCs into a Th1 phenotype [19] (Figure 1).

This dual role of MSCs highlights their potential as both therapeutic agents and carriers for targeted delivery of anti-cancer drugs and genes [20]. For example, MSCs engineered to secrete anti-tumor cytokines [21] or to amplify and deliver oncolytic viruses [22] can directly target tumors while modulating the immune response to enhance therapeutic efficacy. Conversely, the immunosuppressive properties of MSCs can be harnessed to mitigate excessive inflammation and immune-related adverse effects during cancer treatment [23].

## USE OF MSCs AS CARRIERS FOR THERAPEUTIC AGENTS

MSCs can be utilized as carriers for delivering therapeutic agents directly to tumor sites, leveraging their natural ability to home to areas of inflammation and tumorigenesis. This tumor-homing ability allows MSCs to localize within the tumor microenvironment, where they can release anti-cancer drugs, cytokines, or genetic material designed to modulate the immune response and inhibit tumor growth. This strategy not only enhances the concentration of therapeutic agents at the tumor site but also minimizes systemic side effects, making MSCs an attractive vehicle for targeted cancer therapy [24].

## Mechanisms of MSC homing

### Chemokine receptors and ligands

MSCs express various chemokine receptors that enable them to respond to chemokines released by damaged or inflamed tissues. One of the most crucial receptors involved in this process is C-X-C chemokine receptor type 4 (CXCR4), which binds to stromal-derived factor-1 (SDF-1 or CXCL12). SDF-1 is often overexpressed in injured or hypoxic tissues, including tumors, and plays a pivotal role in guiding MSCs to these sites. The CXCR4/SDF-1 axis is a well-documented pathway that facilitates MSC migration to areas requiring repair or immune modulation. For instance, studies have shown that the upregulation of CXCR4 enhances the homing efficiency of MSCs, improving their therapeutic potential in cancer treatment [25, 26].

Another important chemokine receptor expressed by MSCs is C-C chemokine receptor type 2 (CCR2), which interacts with monocyte chemoattractant protein-1 (MCP-1), also known as CCL2. This interaction plays a critical role in the recruitment and migration of MSCs to sites of inflammation and tumors [27]. MCP-1 is a potent chemokine that is highly expressed in the tumor microenvironment and in areas of tissue injury and inflammation [28]. The binding of MCP-1 to CCR2 on MSCs triggers signaling pathways that facilitate their chemotaxis towards these sites. Upon binding to MCP-1, CCR2 activates intracellular signaling cascades, including the phosphatidylinositol 3-kinase (PI3K)/Akt pathway and the mitogen-activated protein kinase (MAPK) pathway [29]. These signaling events result in cytoskeletal reorganization and the directional migration of MSCs towards higher concentrations of MCP-1. Within the tumor microenvironment, MCP-1 is produced by various cells, including tumor cells, stromal cells, and infiltrating immune cells. The elevated levels of MCP-1 create a chemokine gradient that attracts CCR2-expressing MSCs to the tumor site. Once there, MSCs can modulate the immune response, deliver anti-cancer agents, or participate in tissue repair processes.

#### Adhesion molecules

Once MSCs are attracted to a specific site, adhesion molecules facilitate their attachment and integration into the target tissues. MSCs express several adhesion molecules such as integrins (e.g., VLA-4), selectins (e.g., E-selectin), and cell adhesion molecules (CAMs) (e.g., VCAM-1, ICAM-1) [30]. These molecules interact with endothelial cells and extracellular matrix components, aiding in the transmigration of MSCs across the vascular endothelium into the tissue. For example, integrins are crucial for the initial tethering and rolling of MSCs along the endothelium, a key step in their extravasation and subsequent migration into the tumor microenvironment [31].

#### Inflammatory cytokines

Inflammatory environments and tumors produce various cytokines and growth factors that further enhance MSC migration and homing capabilities. Tumor necrosis factor-alpha (TNF-α), interleukins (e.g., IL-1, IL-6), and transforming growth factor-beta (TGF-β) are among the cytokines that can upregulate the expression of chemokine receptors and adhesion molecules on MSCs [32]. These inflammatory mediators not only increase the responsiveness of MSCs to chemotactic signals but also promote their survival and retention within the inflamed or tumor tissue [33].

### Implications for therapy

#### Targeted delivery

The natural ability of MSCs to home to specific sites in the body makes them ideal candidates for targeted therapies. This tumor-homing characteristic is particularly advantageous in cancer treatment, where MSCs can be engineered to deliver therapeutic agents directly to tumor sites. For example, MSCs can be loaded with chemotherapeutic drugs, oncolytic viruses, or genetic material that encodes for anti-cancer proteins. This targeted approach ensures that the therapeutic agents are concentrated at the tumor site, enhancing their efficacy while minimizing systemic exposure and reducing side effects. A recent study demonstrated that MSCs loaded with the chemotherapeutic agent paclitaxel had significantly improved tumor homing due to upregulation of CXCR4 [34]. Wang et al. documented effective targeting and antitumor effects of paclitaxel-loaded MSCs in a glioma tumor model *in vivo* [35]. Another study showed that MSCs loaded with an oncolytic adenovirus selectively targeted and lysed tumor cells in a hepatocellular carcinoma cancer model, further illustrating the therapeutic potential of MSC-based delivery systems [36].

#### Immunomodulation

At sites of inflammation or tumors, MSCs can exert potent immunomodulatory effects. They achieve this by secreting anti-inflammatory cytokines such as interleukin-10 (IL-10), TGF-β, and prostaglandin E2 (PGE2), which can suppress inflammatory responses and modulate immune cell behavior. Additionally, MSCs can interact directly with immune cells, such as T cells, B cells, natural killer (NK) cells, and dendritic cells, to modulate their activity. For example, MSCs can induce the formation of regulatory T cells (Tregs) and inhibit the proliferation of effector T cells, thereby reducing inflammation and promoting immune tolerance [37]. In the context of cancer, MSCs can either promote or inhibit tumor progression, depending on the cytokine milieu and the specific interactions within the tumor microenvironment. Slama et al. highlighted the dual role of MSCs in modulating immune responses, showing that MSCs could either enhance anti-tumor immunity or support tumor growth depending on the dynamic interactions between the different subsets of MSCs, cancer cells, as well as the tumor microenvironment [38].

#### Tissue repair and regeneration

MSCs are not only home to sites of injury but also contribute to tissue repair and regeneration through their ability to differentiate into various cell types and secrete trophic factors. This regenerative capacity makes MSCs valuable for treating a wide range of conditions, from cardiovascular diseases to orthopedic injuries. For instance, MSCs can differentiate into osteoblasts, chondrocytes, and adipocytes, promoting bone and cartilage repair. Additionally, MSCs secrete a variety of growth factors, such as vascular endothelial growth factor (VEGF), fibroblast growth factor (FGF), and insulin-like growth factor (IGF), which promote angiogenesis, cell proliferation, and tissue remodeling [39]. A study by Hoogduijn et al. demonstrated that MSCs isolated from transplanted heart tissue showed an immunophenotype that was characteristic for MSCs and maintained cardiomyogenic and osteogenic differentiation capacity [40]. Importantly, the functional MSCs of donor origin remained present in the transplanted heart for several years after transplantation. Similarly, another study showed that MSCs accelerated wound healing in a skin wound healing model by affecting both dermal fibroblast and keratinocyte migration, along with a contribution to the formation of extracellular matrix [41].

#### Genetic engineering of MSCs

To enhance the therapeutic efficacy of MSCs, they can be genetically engineered to express proteins or genes that boost their immunomodulatory and anti-tumor properties. This genetic modification enables MSCs to deliver more potent therapeutic agents directly to tumor sites, enhancing their overall effectiveness and minimizing systemic side effects. Recent significant advances in genome engineering, particularly through the development and application of clustered regularly interspaced short palindromic repeats (CRISPR) technologies are allowing very precise and effective manipulation of stem cells for therapeutic purposes. One significant achievement is the development of CRISPR interference (CRISPRi) and CRISPR activation (CRISPRa) systems, which allow for precise gene regulation without altering the underlying DNA sequence [42]. These tools have been instrumental in studying gene function in stem cells, enabling researchers to turn specific genes on or off to observe their effects on cell differentiation and development. Another promising new technique is CRISPR-based chromatin remodeling to reprogram cells to a pluripotent state by targeting key loci such as Oct4 or Sox2 [43]. This method of reprogramming cells is crucial for regenerative medicine, as it allows for the generation of pluripotent stem cells from adult cells, which can then be directed to differentiate into any cell type needed for therapy. Additionally, researchers have explored the use of small molecules to enhance the efficiency of CRISPR genome editing in pluripotent stem cells [44]. By improving editing efficiency, these methods can accelerate the development of stem cell-based therapies, making them more feasible for clinical applications. Recently developed CRISPR-GO system to manipulate the 3D genome organization within the nucleus is providing important insights into how spatial genome arrangement affects stem cell function and differentiation [45]. This tool helps in understanding the complex regulatory networks that govern cell fate decisions, which is essential for advancing stem cell biology and therapy. Overall, these promising CRISPR technology-based techniques have significantly advanced the field of stem cell research, offering new tools and methods to manipulate and study stem cells with unprecedented precision. These pioneering genome engineering developments are paving the way for new therapeutic approaches to treat a wide range of diseases by harnessing the potential of stem cells.

##### Secretion of cytokines

MSCs can be genetically modified to secrete therapeutic cytokines, such as interleukin-12 (IL-12) or interferon-beta (IFN-β). IL-12 is a potent immunostimulatory cytokine that promotes the activation of T cells and natural killer (NK) cells, enhancing anti-tumor immunity. Research by Kulach et al. showed that MSCs engineered to express IL-12 significantly inhibited tumor growth in a melanoma model by increasing the number of anticancer M1 macrophages and CD8+ cytotoxic T lymphocytes in tumors of treated mice [46]. MSCs engineered to secrete IFN-β have been shown to home to tumor sites and inhibit tumor growth in preclinical tumor models [47].

##### Pro-apoptotic gene expression

Another strategy involves engineering MSCs to express pro-apoptotic genes that induce cancer cell death. For example, MSCs can be modified to express TRAIL (TNF-related apoptosis-inducing ligand), a protein that induces apoptosis specifically in cancer cells. MSCs engineered to express TRAIL have demonstrated significant anti-tumor effects in preclinical models of glioblastoma and pancreatic cancer [48]. This approach leverages the tumor-homing ability of MSCs to deliver pro-apoptotic signals directly to the tumor site, enhancing the specificity and efficacy of the treatment. Another study by Spano et al. showed that MSCs expressing TRAIL effectively targeted and killed pancreatic cancer cells *in vitro* and *in vivo*, highlighting the potential of pro-apoptotic gene-engineered MSCs in cancer therapy [49].

##### Delivery of oncolytic viruses

One promising approach is to use MSCs to deliver and potentiate oncolytic viruses. Oncolytic viruses are viruses that selectively infect and kill cancer cells while sparing normal cells. However, a major obstacle to this approach is the elimination of the oncolytic viruses by the patient’s immune system. By using MSCs as carriers, these viruses can be protected from the immune system, and delivered directly to the tumor site, where they can replicate and induce tumor cell lysis. For instance, MSCs loaded with an oncolytic measles virus have shown significant anti-tumor effects in a preclinical model of ovarian cancer [50]. The virus-loaded MSCs delivered the virus directly to the tumor, resulting in significant tumor regression and prolonged survival. Both MSCs [51] and Neural Stem Cells (NSCs) [52] were investigated as potential cellular vehicles to deliver therapeutic agents to brain tumors. In a Phase 1 clinical study, Fares et al. reported the treatment of patients with newly diagnosed malignant glioma with oncolytic adenovirus-loaded NSCs [53]. This treatment was safe, as no formal dose-limiting toxicity was reached. Patients had favorable clinical outcomes (in terms of survival), especially in patients with gliomas with unmethylated MGMT promoters, supporting continued investigation of this approach in a phase 2/3 study in a larger cohort of patients with controlled conditions.

## CLINICAL APPLICATIONS OF MSCs IN CANCER THERAPY AND IMMUNOTHERAPY

Several clinical trials have investigated the use of MSCs in cancer treatment. These trials aim to evaluate the safety and efficacy of MSC-based therapies, including their ability to deliver therapeutic agents to tumors and induce an anti-tumor immune response (Table 1).

**Table 1:** Clinical trials investigating the use of MSCs and neural stem cells (NSCs) in cancer treatment

Clinical trial	Therapeutic agent	Cancer type	Phase	Clinical trial ID	Status
MSCs expressing TRAIL	TRAIL	Advanced solid tumors	Phase 1	NCT03298763	Recruiting
MSCs with an oncolytic virus	Oncolytic adenovirus	Solid tumors	Phase ½	NCT01844661	Completed
MSCs with an oncolytic virus	Oncolytic measles virus	Solid tumors	Phase ½	NCT02068794	Recruiting
MSCs expressing interferon beta	Interferon beta	Ovarian cancer	Phase 1	NCT02530047	Completed
MSCs	–	Prostate cancer	Phase 1	NCT01983709	Completed
MSCs expressing IL-12	IL-12	Head and neck cancer	Phase 1	NCT02079324	Completed
MSCs expressing enzymes	Cytosine Deaminase	Glioma	Phase ½	NCT04657315	Completed
MSCs	–	Glioma	Phase 1	NCT05789394	Recruiting
MSCs with an oncolytic virus	Oncolytic adenovirus	Glioma	Phase 1	NCT03896568	Recruiting
NSCs with oncolytic virus	Oncolytic adenovirus	Glioma	Phase 1	NCT03072134	Completed
NSCs with oncolytic virus	Oncolytic adenovirus	Glioma	Phase 1	NCT05139056	Recruiting
NSCs expressing enzymes	Cytosine Deaminase	Glioma	Phase 1	NCT02015819	Completed
NSCs expressing enzymes	Carboxyl Esterase	Glioma	Phase 1	NCT02192359	Completed
NSCs expressing enzymes	Cytosine Deaminase	Glioma	Phase 1	NCT01172964	Completed

These trials have shown promising potential across various cancer types and therapeutic strategies. Several trials are focused on using MSCs engineered to express specific therapeutic agents. For example, MSCs expressing TRAIL (NCT03298763) are being tested in phase 1 trials for advanced solid tumors, while MSCs expressing interferon beta (NCT02530047) have completed phase 1 trials for ovarian cancer. Other trials involve MSCs engineered to express interleukin-12 (IL-12) for head and neck cancer (NCT02079324) and MSCs expressing the enzyme cytosine deaminase for glioma (NCT04657315). Additionally, MSCs are being employed as carriers for oncolytic viruses in multiple clinical trials, aiming to exploit their tumor-homing capabilities to deliver viral therapies directly to tumor sites. Furthermore, Neural Stem Cells (NSCs) have been tested in several phase 1 trials for brain tumors.

These clinical trials exemplify the diverse and innovative approaches being explored to harness the therapeutic potential of MSCs in cancer treatment. By leveraging their natural tumor-homing abilities and combining them with powerful anti-tumor agents, MSCs can deliver targeted and effective treatments to cancer sites. However, these clinical trials have also delineated the difficulties in utilizing this novel approach and provided invaluable insights to support further research into these promising treatment approaches.

## MSC-DERIVED EXOSOMES AS THERAPEUTIC AGENTS

Exosomes, a type of extracellular vesicles (EV) with a size range of 30–150 nm, are secreted by MSCs and serve as critical mediators of cell-to-cell signaling. These vesicles carry a variety of biomolecules, including proteins, lipids, and nucleic acids such as miRNAs, which influence the behavior of target cells. In the context of MSC biology, exosomes are crucial for maintaining homeostasis and mediating tissue repair, immune modulation, and anti-inflammatory responses. Importantly, exosomes are involved in both physiological and pathophysiological activities of MSCs, making them potent agents for therapeutic use. Studies have shown that MSC-derived exosomes can replicate many of the beneficial effects of MSCs themselves, including promoting tissue regeneration, reducing inflammation, and modulating immune responses without the risks associated with stem cell transplantation [54, 55].

Therapeutically, MSC-derived exosomes have emerged as promising candidates in treating a variety of conditions, including cardiovascular diseases, neurological disorders, and cancers. For instance, in cancer therapy, exosomes are being explored as delivery vehicles for anticancer drugs and genetic material due to their natural ability to home to tumor sites and interact with the tumor microenvironment [56, 57]. Recent research also emphasizes the role of MSC exosomes in immunomodulation, with studies showing that they can alter the immune response in diseases such as graft-versus-host disease and autoimmune disorders [58]. The therapeutic potential of MSC-derived EVs, particularly exosomes, continues to grow as more is understood about their roles in cell communication, disease progression, and tissue regeneration, making them a critical focus in regenerative medicine and targeted therapies. This growing field underscores the potential of MSC-derived exosomes as therapeutic tools in diverse clinical settings.

## CHALLENGES AND LIMITATIONS OF MSC THERAPY

### Tumor growth promotion

One of the significant challenges of MSC therapy is the potential for MSCs to promote tumor growth under certain conditions. The immunosuppressive properties of MSCs can sometimes create a tumor-friendly environment, supporting cancer cell survival and proliferation. MSCs secrete various cytokines and growth factors, such as TGF-β, IL-10, and VEGF, which can modulate the immune response and promote angiogenesis, creating a microenvironment that may favor tumor growth [59]. Studies have shown that MSCs can enhance the metastatic potential of certain cancers by promoting epithelial-mesenchymal transition (EMT) and enhancing the invasive properties of cancer cells [60]. This paradoxical effect underscores the complexity of MSC interactions with the tumor microenvironment and necessitates careful consideration and design of MSC-based therapies to avoid unintended pro-tumorigenic effects.

### Variability in MSC preparations

The therapeutic efficacy of MSCs can be influenced by the source of the cells, the isolation and culture methods, and the conditions under which they are expanded. MSCs can be derived from various tissues, including bone marrow, adipose tissue, umbilical cord blood, and placenta, each of which may have distinct biological properties and therapeutic potentials. Additionally, the methods used to isolate and expand MSCs can introduce variability in their characteristics and functions. Differences in culture media, oxygen tension, and passage number can all impact MSC phenotype, viability, and immunomodulatory capabilities [61]. This variability can lead to inconsistent therapeutic outcomes and poses a challenge for the standardization of MSC-based therapies. Establishing robust and reproducible protocols for MSC isolation, culture, and expansion is essential to ensure the consistency and reliability of these therapies in clinical applications [62].

### Safety concerns

There is safety concerns related to the use of genetically engineered MSCs, including the risk of insertional mutagenesis and the potential for uncontrolled cell growth. Genetic modifications, such as the insertion of therapeutic genes, can potentially disrupt endogenous gene function or regulatory elements, leading to insertional mutagenesis and the development of malignancies [63]. Furthermore, the proliferative capacity of MSCs, combined with genetic modifications, raises concerns about the potential for uncontrolled cell growth and tumorigenicity [64]. Rigorous safety evaluations and monitoring are required to address these concerns. Preclinical studies and clinical trials must include comprehensive safety assessments to evaluate the risks of genetic modifications, including long-term follow-up to monitor for potential adverse effects. Ensuring the safety of MSC-based therapies is paramount to their successful translation into clinical practice.

In summary, while MSCs hold great promise for cancer therapy, addressing the challenges and limitations associated with their use is crucial for their safe and effective clinical application. Ensuring consistent cell preparations, mitigating the risk of tumor promotion, and conducting thorough safety evaluations are essential steps in advancing MSC-based therapies.

## FUTURE DIRECTIONS FOR MSC RESEARCH IN CANCER IMMUNOTHERAPY

### Enhancing MSC therapeutic potential

Future research should focus on enhancing the therapeutic potential of MSCs through advanced genetic engineering techniques. One promising approach is the use of CRISPR-Cas9 technology to precisely edit genes involved in immunomodulation and anti-tumor activity. CRISPR-Cas9 allows for targeted gene modifications, enabling researchers to enhance or suppress specific pathways in MSCs to improve their therapeutic efficacy [65]. For instance, MSCs can be engineered to express higher levels of immunostimulatory cytokines, such as IL-12 or IFN-β, or to knock out genes that may promote tumor growth [66].

Combining MSCs with other immunotherapeutic approaches, such as immune checkpoint inhibitors or CAR-T cells, could also improve treatment outcomes. Immune checkpoint inhibitors, which block receptors that inhibit T cell activation, can be used in conjunction with MSCs to enhance anti-tumor immune responses [67]. Similarly, CAR-T cells, which are T cells engineered to express chimeric antigen receptors targeting specific cancer antigens, can be combined with MSCs to create a more robust and targeted anti-cancer therapy. Recent studies have shown that MSCs can improve the anticancer functions of CAR-related products. Thus, MSCs can be used as a biological vehicle for CARs, as CAR-MSCs might be able to overcome the flaws of cellular immunotherapy [68]. The synergy between MSCs and these advanced immunotherapies holds great promise for improving the efficacy of cancer treatments.

### Understanding MSC-tumor interactions

A deeper understanding of the interactions between MSCs and the tumor microenvironment is essential for optimizing their use in cancer immunotherapy. Research should aim to elucidate the molecular mechanisms underlying MSC migration, homing, and immunomodulatory effects in different types of cancers. Studies have shown that MSCs interact with various components of the tumor microenvironment, including cancer cells, immune cells, and stromal cells, through complex signaling networks involving cytokines, chemokines, and growth factors.

For example, the role of the CXCR4/SDF-1 axis in MSC homing to tumor sites has been well documented, but further research is needed to understand how this pathway interacts with other signaling mechanisms in the tumor microenvironment [69]. Additionally, the immunomodulatory effects of MSCs can vary depending on the specific cytokine milieu and cellular context within the tumor. Understanding these interactions can help identify strategies to enhance the anti-tumor effects of MSCs while minimizing potential pro-tumorigenic activities [38].

### Personalized MSC therapies

Developing personalized MSC therapies tailored to the specific characteristics of a patient’s tumor and immune system could enhance the efficacy and safety of MSC-based treatments. This approach would involve profiling the tumor microenvironment and designing MSC therapies that are optimized for individual patients. Personalized MSC therapies could be developed by analyzing the genetic and molecular profiles of tumors and the immune landscape of the patient to identify the most effective therapeutic targets and strategies.

Advances in single-cell sequencing and other high-throughput technologies have enabled detailed characterization of the tumor microenvironment at a cellular level, providing insights into the heterogeneity and dynamics of tumor-immune interactions [70]. By integrating these data with patient-specific clinical information, researchers can design MSC therapies that are tailored to the unique characteristics of each patient’s cancer. Personalized MSC therapies have the potential to improve treatment outcomes by maximizing therapeutic efficacy and minimizing adverse effects [71].

## SURPRISING TWISTS IN THE USE OF MSCs FOR CANCER IMMUNOTHERAPY

The following key considerations underscore the importance of a nuanced approach to using MSCs in cancer immunotherapy, balancing their therapeutic potential with the complexities of their interactions within the tumor microenvironment.

### Dual role in tumor progression

While the MSCs can support anti-tumor immune responses and enhance the delivery of therapeutic agents to tumors, they can also, under certain conditions, promote tumor growth and metastasis. This paradoxical behavior necessitates a comprehensive understanding to effectively harness MSCs in cancer therapy. For instance, MSCs can enhance tumor cell proliferation and invasion by secreting growth factors and cytokines that modulate the tumor microenvironment [72, 73].

### Tumor homing abilities

The inherent ability of MSCs to home to tumor sites presents both an advantage and a challenge. Initially viewed as a purely beneficial trait for delivering therapies directly to tumors, the underlying mechanisms – driven by chemokine signaling and the inflammatory tumor microenvironment – reveal complexities in accurately predicting and controlling MSC migration. The CXCR4/SDF-1 axis, for example, is pivotal in this process but requires precise modulation to ensure targeted delivery [25, 26, 69].

### MSC-induced immunosuppression

MSCs can suppress immune responses, which is advantageous for treating inflammatory diseases but poses significant challenges in cancer therapy. MSCs can inhibit T cell proliferation and natural killer (NK) cell activity, potentially enabling tumors to evade immune surveillance. This immunosuppressive capability necessitates the careful design of MSC-based therapies to avoid inadvertently protecting the tumor [14, 15].

### Unexpected differentiation potential

MSCs are known for their multipotency, but their ability to differentiate into various cell types within the tumor microenvironment can have unintended consequences. For example, MSCs can differentiate into carcinoma-associated fibroblasts (CAFs), which support tumor growth and metastasis, complicating their therapeutic use. This differentiation potential underscores the need for strategies to control MSC fate within the tumor microenvironment [74, 75].

### MSC-mediated delivery of oncolytic viruses

Using MSCs to deliver oncolytic viruses directly to tumors is a novel and promising approach. However, the interactions between MSCs, the immune system, and oncolytic viruses can lead to unexpected outcomes, such as enhanced anti-tumor immunity or rapid clearance of the therapeutic viruses before they can effectively act on the tumor. This complexity requires careful optimization of MSC and oncolytic virus combinations [22, 76].

### Heterogeneity and variability

MSCs derived from different tissue sources (e.g., bone marrow, adipose tissue, umbilical cord) exhibit varying degrees of efficacy and behavior in cancer therapy. This heterogeneity introduces challenges in standardizing MSC-based treatments and predicting therapeutic outcomes. Standardization of isolation and culture methods is crucial to ensure consistency and reliability [77].

### Interaction with the tumor microenvironment

The tumor microenvironment (TME) can influence MSC behavior in unpredictable ways. For instance, the hypoxic conditions within the TME can enhance MSC survival and therapeutic efficacy but can also promote MSC differentiation into pro-tumorigenic phenotypes [78]. This necessitates a nuanced approach in therapeutic planning and execution to mitigate pro-tumorigenic effects while leveraging therapeutic benefits.

## CONCLUSIONS AND PROSPECTS

MSCs hold great promise as a therapeutic tool in cancer immunotherapy due to their immunomodulatory properties, tumor-homing abilities, and potential as carriers for delivering therapeutic agents. MSCs can be engineered to stably express various antitumor agents, overcoming the limitations of conventional therapies. The future of MSC therapy involves a deeper understanding of fundamental stem cell mechanisms, the interplay between normal and cancer stem cells, and the application of advanced engineering techniques to improve efficacy.

Given the enormous complexity of cancer development, combination therapies will be necessary to achieve durable therapeutic benefits. Engineered MSC products, with their off-the-shelf nature, hold promise as components of comprehensive treatment regimens that include established cancer treatments, such as surgery, radiation, chemotherapy, and targeted therapies, as well as emerging cancer immunotherapies, such as immune checkpoint inhibitors, oncolytic viruses, cancer vaccines, and adoptive cellular therapies. The versatility of engineered MSC products and their ability to be tailored and scaled for specific cancer types make them a promising addition to the oncology arsenal. As the field continues to evolve, maintaining a cautious and meticulous approach is crucial to ensure the safe and effective translation of stem cell-based therapies into clinical settings.

MSCs improve the anticancer efficacy of the oncolytic virotherapy in various ways. They act as a reproduction site for oncolytic viruses (OVs), generating more virions to support more effective virotherapy. MSCs’ tumor tropism and immunosuppressive activity enable the OVs to target the cancer sites specifically, increasing viral spread and survival. Furthermore, MSCs generate cytokines that attract immune cells to the tumor site, enhancing local anticancer immune responses and transforming the tumor microenvironment from immunosuppressive to immunostimulatory. Integrating MSCs with more effective OVs is a logical step towards enhancing therapeutic outcomes. Currently, several ongoing clinical trials are exploring the use of OV-loaded MSCs for cancer therapy, offering a wide range of promising combination approaches.

Despite the promising potential of MSCs, clinical trials on their use in cancer therapy have yielded mixed results. This variability may be due to the tumor immune microenvironment’s effects, where immune cells are inhibited by various factors, creating a conducive environment for tumor growth. MSCs influence tumor immune regulation by enhancing or suppressing immune activation, thereby affecting therapeutic outcomes differently across tumor types. Therefore, extensive MSC characterization will be necessary to identify MSCs with strong anti-cancer potential.

In summary, MSCs offer promising potential applications in cancer therapy. However, their therapeutic effects vary across different tumor types, necessitating a deeper understanding of their immunomodulatory roles and interactions with the tumor microenvironment. While challenges and limitations exist, ongoing research and clinical trials continue to advance our understanding and application of MSCs in cancer treatment. By addressing these challenges and harnessing the full potential of MSCs, we can develop more effective and personalized cancer therapies, offering new hope for patients with difficult-to-treat malignancies.
